# *Giardia duodenalis* and *Entamoeba* spp.: Coprological and antibody–based approaches across farm animals in Egypt

**DOI:** 10.1186/s12917-026-05801-4

**Published:** 2026-08-14

**Authors:** Nagwa I. Toaleb, Kadria N. Abdel Megeed, Dina Aboelsoued

**Affiliations:** Parasitology and Animal Diseases Department, Veterinary Research Institute, National Research Centre, El Buhouth St., Dokki, Giza, Egypt

**Keywords:** Microscopy, Sandwich ELISA, PCR, Protein-A-Sepharose Affinity, Farm Animals

## Abstract

*Giardia duodenalis* (syn. *G. lamblia, G. intestinalis*) and *Entamoeba* spp. are enteric protozoans causing gastrointestinal illnesses in humans and animals worldwide. This study aimed to determine the prevalence of *Giardia* and *Entamoeba* infections in farm animals in Egypt, and to develop an in-house sandwich ELISA for their coproantigen detection. In this study, a total of 2674 fecal samples were collected from buffaloes (*N* = 875), cattle (*N* = 770), sheep (*N* = 520), and goats (*N* = 509) from different age groups across five governorates. All samples were examined using conventional microscopical methods. Using microscopy, *Giardia* and *Entamoeba* infections were detected in 626/2674 (23.4%) and 457/2674 (17.1%) of samples, respectively, and sheep showed the highest infection rates by both parasites (44.6% and 28.8% for *Giardia* and *Entamoeba,* respectively). Representative isolates were molecularly characterized, to confirm parasite identity prior to antigen preparation, targeting *β-giardin* (*bg*) and small subunit ribosomal RNA (*ssu rRNA*) gene, confirmed the presence of *G. duodenalis* (GenBank: OQ388331.1, OQ388332.1, OR029977.1, OR029976.1, OR029974.1, OR029975.1 and PP316110.1) and *E. histolytica* (GenBank: OR016432.1). Antigens were prepared separately from these isolates, and the polyclonal antibodies (pAbs) were prepared in rabbits and purified using Protein-A-Sepharose affinity columns. Then, Anti‑*Giardia* IgG pAbs and Anti-*Entamoeba* IgG pAbs in-house sandwich ELISAs were tested separately on all fecal samples revealing infection rates of 45.2% (1208/2674) for *Giardia* sp. and 30.1% (805/2674) for *Entamoeba* sp. with high ability to detect *Giardia* (*χ*^*2*^ = 281.2, *p* < *0.001*) and *Entamoeba* (*χ*^*2*^ = 125.6, *p* < *0.001*) species compared to microscopy. In conclusion, the developed sandwich ELISA in our study represents a sensitive and reliable diagnostic tool for the detection of *Giardia* and *Entamoeba* infections in farm animals. Its application in routine surveillance along with complementary molecular confirmation could help to improve epidemiological accuracy and assess zoonotic risks.

**Supplementary Information:**

The online version contains supplementary material available at 10.1186/s12917-026-05801-4.

## Introduction

Intestinal protozoan infections caused by *Giardia duodenalis* (syn. *G. lamblia, G. intestinalis*) and *Entamoeba* spp. have considerable importance in veterinary medicine due to their wide distribution in farm animals, impact on productivity, and zoonotic relevance [37, 69, 76]. These parasites infect a broad range of farm animals, including buffaloes, cattle, sheep, and goats, through ingestion of infective cysts from contaminated feed, water, or environment [23, 63].

Giardiasis causes enteric manifestations in livestock related to diarrhea, which severity is age-dependent, being more prominent in animals younger than 1 month old until they are six months of age [63]. In addition, *G. duodenalis* is recognized as one of the most prevalent enteric protozoa in livestock worldwide, induced by decreased absorption of nutrients, fluids and electrolytes leading to immense economic losses, especially in meat- and milk-oriented cattle farms, which are also attributed to high treatment costs and calf mortality [64, 69, 76]. Also, the circulation of zoonotic assemblages of *G. duodenalis* reinforces their relevance within the One-Health framework, especially in Egypt [6, 24, 50]. Similarly, *Entamoeba* species are reported in livestock, however, most infections in farm animals are asymptomatic or of unclear pathogenic significance and may involve non-pathogenic species such as *E. dispar* and *E. coli*, which are morphologically indistinguishable under light microscopy [7]. Under certain conditions such as co-infections, stress or poor management practices, *E. histolytica* could cause enteric disturbances, decreased weight gain and decreased productivity [80]. Close contact between humans and livestock increases the potential for zoonotic transmission of these parasites and environmental contamination [15, 79].

The incubation period is 9–15 days for giardiasis [37, 47] and 1–4 weeks for amoebiasis [18, 57]. Diagnosis of giardiasis and amoebiasis is based on the identification of *Giardia* cysts or motile trophozoites and *Entamoeba* cysts in fecal samples under microscope [8], as well as the detection of their antigens in fecal samples [40]. Accurate diagnosis of these protozoa represents a major challenge in epidemiological and clinical studies as conventional microscopical techniques are still widely used in field and laboratory settings due to their low cost and simplicity. However, these methods have limited sensitivity which could be primarily due to intermittent cyst shedding and low parasite burden [82].

In farm animal populations, evaluation of diagnostic tools remain limited and most available studies focused on non-livestock hosts, leaving a gap in understanding the performance of combined approaches such as microscopy versus sandwich ELISA in ruminants. Given the importance of accurate diagnosis for surveillance studies, control strategies, and zoonotic risk assessment, this study was constructed to evaluate the diagnostic performance of an in-house diagnostic approach (Sandwich ELISA) in the detection of *Giardia* and *Entamoeba* antigens under field conditions. Molecular characterization of representative isolates was performed solely to confirm parasite identity prior to antigen preparation, while other gastrointestinal parasites were evaluated to investigate mixed infections and assess assay specificity.

## Materials and methods

### Study area

This cross-sectional study was conducted to monitor the prevalence of *Giardia* and *Entamoeba* spp. in newborn and adult farm animals in Cairo (30°02′N, 31°13′E), Giza (29° 58′ 27.00″ N, 31° 08′ 2.21″ E), Kafr ElSheikh (31° 18′ 0″ N, 30° 55′ 48″ E), El-Beheira (30.61°N 30.43°E), and Qalyubia (30.41°N 31.21°E) governorates, Egypt, during the period between January 2023 to December 2024.

### Sample size

Sample size was calculated as described by Daniel [19] using the formula:

$${\boldsymbol{n}}=\frac{{{\boldsymbol{Z}}}^{\boldsymbol2}{\boldsymbol{p}}(1-{\boldsymbol{p}})}{{{\boldsymbol{d}}}^{2}}$$

where: *n* = required sample size, *Z* = appropriate percentage for the standard deviation (SD) with expected confidence interval CI (95%), *p* = estimated prevalence, and *d* = anticipated absolute accuracy (5%). The expected prevalence values were selected based on previous epidemiological studies and recommendations by Thrusfield et al. [71]. We used 25% anticipated prevalence for *Giardia* spp. and 20% for *Entamoeba* spp., based on previous epidemiological studies in ruminants under similar conditions. Although the minimum sample size required was 288 animals, the sample size was substantially increased to enhance statistical power, improve the precision of infection rates, and enable stratified analysis across host species. Accordingly, a total of 2674 animals were included in the study, comprising 875 buffaloes, 770 cattle, 520 sheep, and 509 goats.

### Animals and sample collection

A total number of 2674 fecal samples were collected from farm animals, 390 buffalo calves, 420 cattle calves (ages ranged from 1 day to 12 months), 160 lambs (less than 12 month), 158 goat kids (less than 9 weeks), 485 adult buffaloes, 350 adult cattle, 360 sheep, and 351 goats, from different small-scale farms and local farmers in Cairo, Giza, Kafr ElSheikh, El-Beheira, and Qalyubia governorates, Egypt. Fecal samples were collected directly from the rectum of each animal, with permission from its owner, using sterile plastic gloves, kept in separate, labelled, clean containers, transferred to the Immunology and Parasitology Laboratory, National Research Centre (NRC), Egypt, and stored at 4 °C. Samples were processed and examined on the day of collection.

### Parasitological examination

#### Macroscopical examination

Fecal consistency was scored for each sample either normal, soft, or watery. The presence of blood, mucus, or other unusual components and color abnormalities were noted as described by Zajac et al. [83] and Saleh et al. [63].

#### Microscopical examination

##### Direct wet smear

Firstly, fecal samples were filtered by a double layer gauze to remove debris and large particles. Wet mounts were prepared from fresh feces (about 15–20 g/sample) for the detection *Giardia* and *Entamoeba* spp. cysts, either unstained or iodine stained (1.5‒2% Lugol’s solution). Positive smears were identified using light microscope (LEICA Imaging Systems Ltd., England, 40 ×) according to Elmahallawy et al. [25] and Al-Dabbagh et al. [10].

##### Modified Ziehl–Neelsen (MZN) staining

MZN staining technique was performed to identify concurrent *Cryptosporidium* infections, which were included to evaluate mixed gastrointestinal infections and to assess the analytical specificity of the developed sandwich ELISA, according to Henriksen and Pohlenz [38]. Stained slides were examined under a light microscope (LEICA Imaging Systems Ltd., England, 100 × oil immersion).

##### Sedimentation and flotation techniques

All fecal samples were examined by sedimentation and flotation (using saturated salt solution, specific gravity 1.020) techniques as mentioned by Kaufmann [44]. Detected parasite ova, (oo)cysts, trophozoites and larvae were identified based on morphological features under microscope (LEICA Imaging Systems Ltd., England, 40 ×) according to Soulsby [68].

### Preparation of Sandwich ELISA for diagnosis of *Giardia* and *Entamoeba* spp.

#### Parasite samples

Sixty *Giardia-*positive fecal samples collected from buffaloes, cattle, sheep and goats (15 samples each) and 25 *Entamoeba-*positive fecal samples collected from sheep all reared by local farmers in Cairo and Giza governorates, Egypt, were provided by the Research Project (13010124) funded by NRC. *Giardia* cysts were isolated according to Roberts-Thomson et al. [61] with minor adaptations according to Alvarado and Wasserman [12]. While cysts of *Entamoeba* were isolated from positive fecal samples following the procedure of Walderich et al. [78].

#### Identification of cysts’ isolates

Representative isolates selected for antigen preparation were molecularly confirmed to ensure the identity of the parasite antigens used for immunization and assay development.

##### Genomic DNA extraction

*Giardia* and *Entamoeba*-heavily infected microscopy-positive buffalo, cattle and sheep samples, showing abundant, morphologically typical cysts to ensure sufficient DNA yield for sequencing and phylogenetic analysis, were subjected to DNA extraction using a DNA Extraction Kit (QIAamp DNA Stool MiniKit, Cat. no.: 51504, USA) according to the manufacturer’s procedure. DNA concentration was assessed by a microvolume spectrophotometer (Q9000, Quawell, USA) and stored at − 20°C.

##### PCR

*Giardia* DNA was identified by PCR targeting 753 bp the *β-giardin* (*bg*) gene using primers G7: AAG CCC GAC GAC CTC ACC CGC AGT GC and G759: GAG GCC GCC CTG GAT CTT CGA GAC GAC [14]. The amplification assays were conducted using Emerald Amp GT mastermix™ (Takara) in a thermal Cycler (BIO-RAD, Singapore) with an initial denaturation of 94 °C for 5 min, 35 cycles including denaturation at 94 °C for 30 s, annealing at 50 °C for 40 s, and extension at 72 °C for 45 s, and final extension at 72 °C for 10 min. *Entamoeba* DNA was identified by PCR targeting 439 bp the small subunit ribosomal RNA (*ssu rRNA*) gene using primers EHF: AAG CAT TGT TTC TAG ATC TGA G and EHR: AAG AGG TCT AAC CGA AAT TAG [55]. The cycling conditions were the same as *Giardia* PCR except the annealing temperature was 48 °C for 40 s. Final PCR products were visualized by a Molecular Imager (BIO RAD, Singapore) in agarose gel electrophoresis (1.5%) stained with RedSafe (Intron Biotechnology, Republic of Korea), with a 100 bp ladder (QIAGEN, USA).

##### Sequencing and phylogenetic analysis

PCR products were purified by a Gel Extraction Kit (GeneDireX, USA) according to manufacturer’s instructions. Then, the purified products were sequenced by sequencing kit (Big Dye Terminator V3.1 Cycle, Perkin-Elmer, USA) with ABI 3130 automated sequencer (Applied Biosystems, USA). Sequences were corrected by ChromasPro 1.7 Software (Technelysium Pty Ltd., Australia) and compared to others available in GenBank with the nucleotide BLAST and submitted to GenBank. Multiple sequences alignment was performed using CLUSTAL W 1.83 of MegAlign module of Lasergene DNAStar Software Pairwise. Phylogenetic analysis was done with maximum likelihood according to Kumar et al. [49] using MEGA7 software.

#### Preparation of crude antigens of *Giardia* (CGAg) and* Entamoeba* (CEAg)

Isolated *G. duodenalis* and *E. histolytica* cysts (2 × 10^5^—3 × 10^5^ cysts in 1 mL) were separately suspended in 10 mM phosphate buffered saline (PBS, pH = 6.8) as described by Green et al. [34], subjected to many freezing and thawing cycles, and sonicated on ice for 12 cycles/10 s (Sonics Vibra Cell VCX750 Sonicator; Sonics & Materials, Inc., USA), for disrupting the cell wall [36]. Then centrifuged at 12,000 × *g* for 20 min at 4 °C, and the supernatant was assayed for protein content estimation by the method described by Lowry et al. [51]. Supernatants were represented as crude *Giardia* (CGAg) and *Entamoeba* (CEAg) antigens separately and stored at ‒20 °C until needed.

#### Preparation of anti-*Giardia *IgG and anti-*Entamoeba* IgG polyclonal antibodies

Eight healthy bred New Zealand white rabbits (weight: 1.5‒2 kg) were obtained from local supplier and examined before starting experiments to ensure they were parasite-free by fecal examination for three consecutive days using flotation, sedimentation [68] and MZN staining techniques [38]. All rabbits were kept at the animal house in NRC under strictly sanitized laboratory conditions with free access to food and water. Each rabbit was immunized by the crude antigens: CGAg and CEAg, 4 rabbits each, as described by Tendler et al. [70] with some modifications according to Maher et al. [52]. Blood samples were collected from rabbits at week intervals from the ear vein for two months. Sera were separated and stored in aliquots at − 20 °C.

#### Evaluation of specific antibody responses

The produced CGA IgG pAbs and CEAg IgG pAbs were tested by indirect ELISA as described by Toaleb et al. [73] with some modifications. Briefly, ELISA microtiter plates were coated with 100 μL/well of CGAg and CEAg in carbonate buffer, pH = 9.6, separately, and incubated at 4 °C overnight. The plates were washed with PBS/Tween (pH = 7.4), then blocked with 0.1% bovine serum albumin (BSA, Sigma Aldrich). After 2 h of incubation at 37 °C, 100 μL of diluted hyperimmune rabbit sera (Anti-CGA pAbs IgG and CEAg pAbs IgG) were added separately, and the plates were incubated for 1.5 h at 37°C. After washing, 100 μL of anti-rabbit IgG horseradish peroxidase conjugate (Sigma Aldrich) was added and the plates were incubated at 37 °C for 1 h. The substrate O-phenylenediamine-dihydrochloride (OPD, Sigma Aldrich) was added and the plates were incubated for 15 min in the dark at room temperature (RT). The enzyme reaction was stopped with H_2_SO_4_. Absorbances were measured at 450 wavelengths using a microplate ELISA reader (Bio-Tek Instruments, ELx808 UV, USA).

#### Purification of anti-*Giardia* IgG pAbs and anti- *Entamoeba* IgG pAbs

Rabbit polyclonal anti-*Giardia* and anti-*Entamoeba* cysts antibodies were purified from hyperimmune rabbit sera by ammonium sulfate precipitation, as described by Farid [27] and Abd El Hafez et al. [1] and a Protein-A-Sepharose column (Sigma, St. Louis, Missouri, USA) according to Aboelsoued et al. [3]. The protein concentrations of the purified anti-*Giardia* IgG pAb and anti-*Entamoeba* IgG pAb were measured according to Lowry et al. [51].

#### Labeling of anti-*Giardia *IgG pAbs and anti-*Entamoeba* IgG pAbs with Horseradish Peroxidase (HRP) enzyme

Labeling of anti-*Giardia* IgG pAbs and anti-*Entamoeba* IgG pAbs with HRP enzyme were performed following the method described by Avrameas [13] and Toaleb et al. [73]. After labelling, the HRP conjugate anti-*Giardia* IgG pAbs and anti-*Entamoeba* IgG pAbs (supernatants) were stored at ‒20 °C in small aliquots until further use.

#### Sandwich ELISA

Fecal samples from all animals were prepared for sandwich ELISA by mixing one part of fecal sample (50 mg) into PBS/Tween buffer. The homogeneous suspensions were centrifuged for 3 min at 12,000 × *g*, and the clear supernatants were stored at ‒20 °C until use. After standardization of sandwich ELISA using checkerboard titration, the selected concentrations of coating (anti‑*Giardia* IgG and anti-*Entamoeba* IgG polyclonal antibodies) and HRP conjugated antibodies were 1/100 and 1/50 μg/mL, respectively. Sandwich ELISA was performed according to Maher et al. [52] and Aboelsoued et al. [3]. Briefly, the plates were coated with 100 μL/well of IgG polyclonal antibodies (anti‑*Giardia* IgG and anti-*Entamoeba* IgG), separately, incubated at 4 °C overnight; washed 3 times in PBS/Tween (pH = 7.2), blocked with blocking buffer and incubated in RT for 2 h. After that 100 μL/well of the prepared fecal samples were pipetted in triplicates and incubated at 37 °C for 1.5 h. Peroxidase-conjugated pAbs (100 μL/well) were added separately and incubated at 37 °C for 1 h. OPD was added, and the plates were incubated for 30 min at RT in the dark; then the reaction was stopped by H_2_SO_4_ (50 μL/well). The absorbances were measured at 450 nm using an ELISA reader (Bio-Tek Instruments, ELx808 UV, USA). The ELISA plate was coated with purified anti-*Giardia* IgG pAbs and anti-*Entamoeba* IgG pAbs and the prepared anti-*Giardia* IgG pAbs HRP and anti-*Entamoeba* IgG pAbs HRP were used separately as conjugates.

#### Validation of the developed Sandwich ELISA

The analytical performance of the developed sandwich ELISA was evaluated through a multistep validation process. Optimal concentrations of coating antibodies and HRP-conjugated antibodies were established using checkerboard titration. The assay cut-off value was determined as the mean optical density (OD) of parasite-free negative fecal samples plus three standard deviations (3 SD; [2]). Diagnostic performance was evaluated using microscopy-positive and PCR-confirmed representative samples, positive *G. duodenalis* infected sheep fecal samples (OQ388332.1 and OQ388331.1; *N* = 30) and *E. histolytica* infected sheep fecal samples (OR016432.1; *N* = 24), together with parasite-free negative controls. Analytical specificity was assessed by testing animal fecal samples naturally infected with *C. parvum* (OR029973.1; *N* = 20), *Fasciola gigantica* (*n* = 15), *Strongyloides* sp. (*N* = 12), and *Eimeria* sp. (*N* = 12), compared to non-infected fecal samples free from *Giardia* and *Entamoeba* cysts’ antigens and free of other parasites infections (healthy animals, *N* = 22) as confirmed by microscopic examination and PCR to determine potential cross-reactivity. Diagnostic sensitivity, specificity, positive predictive value (PPV), negative predictive value (NPV), diagnostic efficiency, and receiver operating characteristic (ROC) curve analysis with area under the curve (AUC) were calculated to evaluate assay performance according to Parikh et al. [58].

### Statistical analysis

Statistical analyses were performed using SPSS Software V.19 for Windows (IBM, NY, USA). Results of microscopical examination and sandwich ELISA were analyzed using Chi-square test. The diagnostic accuracy parameters of Sandwich ELISA were evaluated using SPSS software. *p* < *0.05* was considered statistically significant.

## Results

### Parasitological examination

As shown in Tables 1 and 2, we detected single parasitic infections in 1558 of 2674 (58.3%), and mixed infections (co-infections) by two or more GI parasite species were detected in 610 of 2674 (22.8%). Regarding *Giardia* spp., sheep had the highest infection rate (44.6%, 232/520) followed by goats (30.1%, 153/509), cattle (18.4%, 142/770), and buffaloes (11.3%, 99/875) and the total infection rate was 23.4% of all farm animals examined (Table 1). Whereas *Entamoeba* spp., infection was detected in 17.1% of farm animals; 150 of 520 (28.8%) in sheep, 116 of 509 (22.8%) in goats, 118/875 (13.5%) in buffaloes and 73/770 (9.5%) in cattle fecal samples (Table 1). A clear age-related trend was observed across protozoan infections (*Giardia, Entamoeba, Cryptosporidium* and *Eimeria*) were more prevalent in younger animals, particularly those under 6 months of age. Conversely, helminth infections such as *Fasciola* spp. and *Strongyloides* spp. were more frequently detected in older animals. These differences were statistically significant (*P* < *0.05*), indicating that age is an important risk factor for gastrointestinal (GI) parasitic infections (Supplement. Table 1). Furthermore, prevalence of mixed infections of *Giardia* and *Entamoeba* spp. with other protozoans and helminth parasites in examined farm animals (Table 2).

**Table 1:** *Giardia* and *Entamoeba* species infection rates according to host species and age group of animals

Parasite species		*Giardia*	*Entamoeba*
Animal species/age			
Buffaloes	** < 4 months (*N* = 88)**	34 (38.6%)	27 (30.7%)
	**4–6 months (*N* = 122)**	29 (23.8%)	25 (20.5%)
	**6–12 months (*N* = 180)**	16 (8.9%)	22 (12.2%)
	** ≥ 1 year (*N* = 485)**	20 (4.1%)	44 (9.1%)
	**Sub-total (*N* = 875)**	**99 (11.3%)**	**118 (13.5%)**
Cattle	** < 4 months (*N* = 88)**	35 (39.8%)	26 (29.5%)
	**4–6 months (*N* = 122)**	56 (45.9%)	33 (27%)
	**6–12 months (*N* = 190)**	28 (14.7%)	9 (4.7%)
	** ≥ 1 year (*N* = 350)**	23 (6.6%)	5 (1.4%)
	**Sub-total (*N* = 770)**	**142 (18.4%)**	**73 (9.5%)**
Sheep	**Lambs (*N* = 160)**	84 (52.5%)	61 (38.1%)
	**Sheep (*N* = 360)**	148 (41.1%)	89 (24.7%)
	**Sub-total (*N* = 520)**	**232 (44.6%)**	**150 (28.8%)**
Goats	**Goat kids (*N* = 158)**	70 (44.3%)	46 (29.1%)
	**Goat (*N* = 351)**	83 (23.6%)	70 (19.9%)
	**Sub-total (*N* = 509)**	**153 (30.1%)**	**116 (22.8%)**

**Table 2:** Prevalence of mixed infections of *Giardia* and *Entamoeba* spp. with different parasites in examined farm animals

Parasites	*Giardia* + *Entamoeba*	*Giardia* + *Cryptosporidium*	*Entamoeba* + Cryptosporidium	*Entamoeba* + *Eimeria*	*Giardia* + *Toxocara Vitulorum*	*Giardia* + *Strongyloides*	*Entamoeba* + *Fasciola*	*Entamoeba* + Strongyloides
Animals								
Buffaloes *N* = 875	23 (2.6%)	27 (3.1%)	21 (2.4%)	19 (2.2%)	19 (2.2%)	11 (1.3%)	14 (1.6%)	15 (1.7%)
Cattle *N* = 770	33 (4.3%)	42 (5.5%)	15 (1.9%)	12 (1.6%)	21 (2.7%)	15 (1.9%)	8 (1%)	11 (1.4%)
Sheep *N* = 520	38 (7.3%)	18 (3.5%)	20 (3.8%)	17 (3.3%)	0	16 (3.1%)	13 (2.5%)	19 (3.7%)
Goats *N* = 509	55 (10.8%)	37 (7.3%)	7 (1.4%)	10 (2%)	0	27 (5.3%)	9 (1.8%)	18 (3.5%)

### Phylogenetic analyses and molecular identification of *Giardia* and *Entamoeba* isolates

**Fig. 1:**
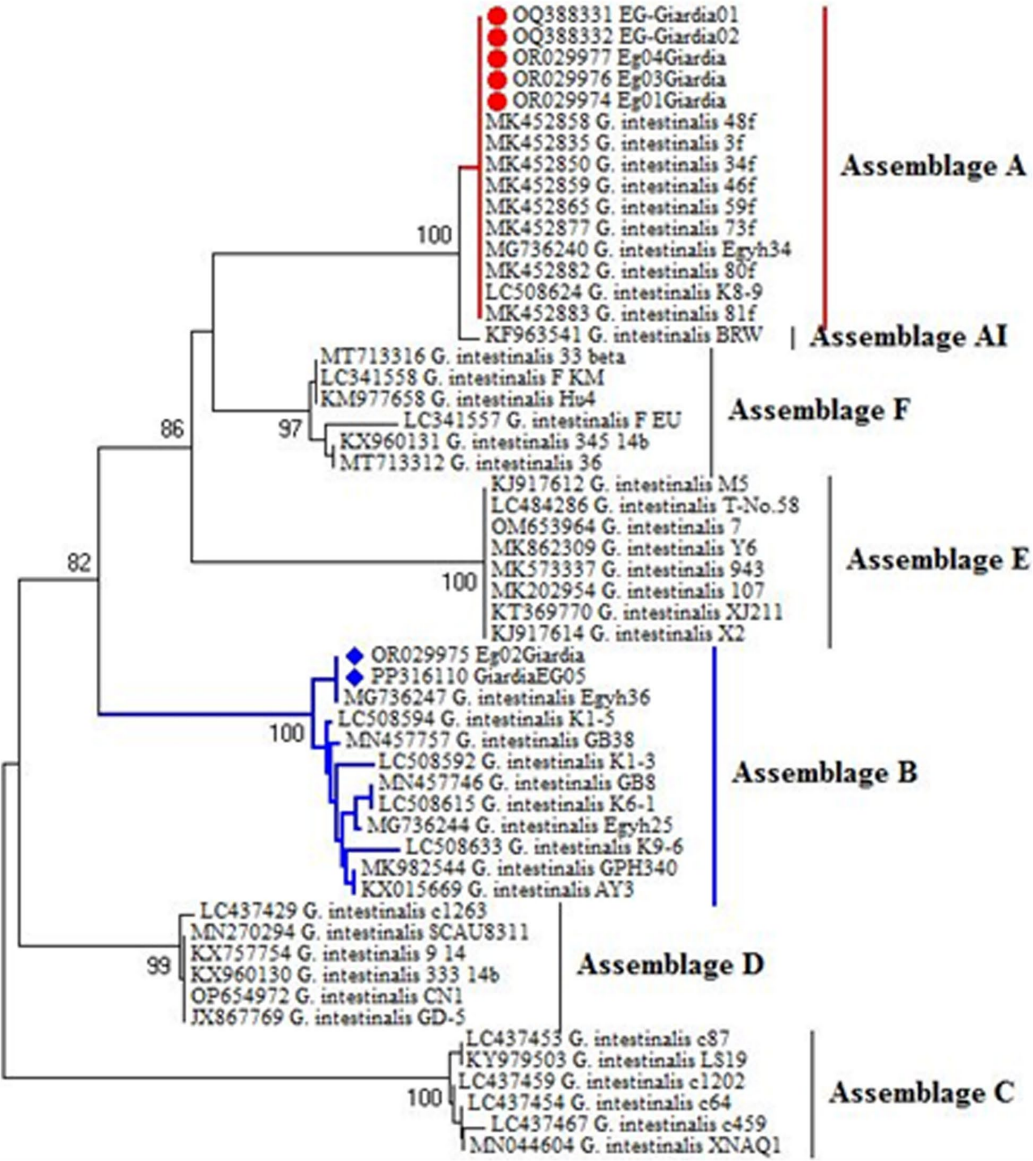
Phylogenetic analysis using the maximum likelihood method based on *β-giardin* gene for *Giardia* sp. Our obtained isolates clustered in well-supported branches with other *Giardia* references, are highlighted (red circle: *G. duodenalis* Assemblage A, blue diamond: *G. duodenalis* Assemblage B)

*Giardia* DNA was detected in the fecal samples of heavily infected buffaloes, cattle, sheep and goats. The nucleotide BLAST search revealed seven genotypes, and their sequences were submitted to GenBank. These genotypes were *G. duodenalis* in sheep from Giza (GenBank: OQ388331.1), sheep from Cairo (GenBank: OQ388332.1), buffalo from Cairo (GenBank: OR029977.1), buffalo from Giza (GenBank: OR029976.1), cattle from Giza (GenBank: OR029974.1), cattle from Cairo (GenBank: OR029975.1) and goat from Giza (GenBank: PP316110.1). Phylogenetic analysis based on the *β-giardin* gene demonstrated that all study isolates clustered within the zoonotic *G. duodenalis* Assemblage A with strong bootstrap support (Fig. 1). The isolates showed high sequence similarity to previously reported human and livestock isolates from Egypt and other geographical regions, indicating the circulation of genetically related strains among different hosts. Assemblage B (highlighted in blue) shows high intra-group diversity (Fig. 1).

**Fig. 2:**
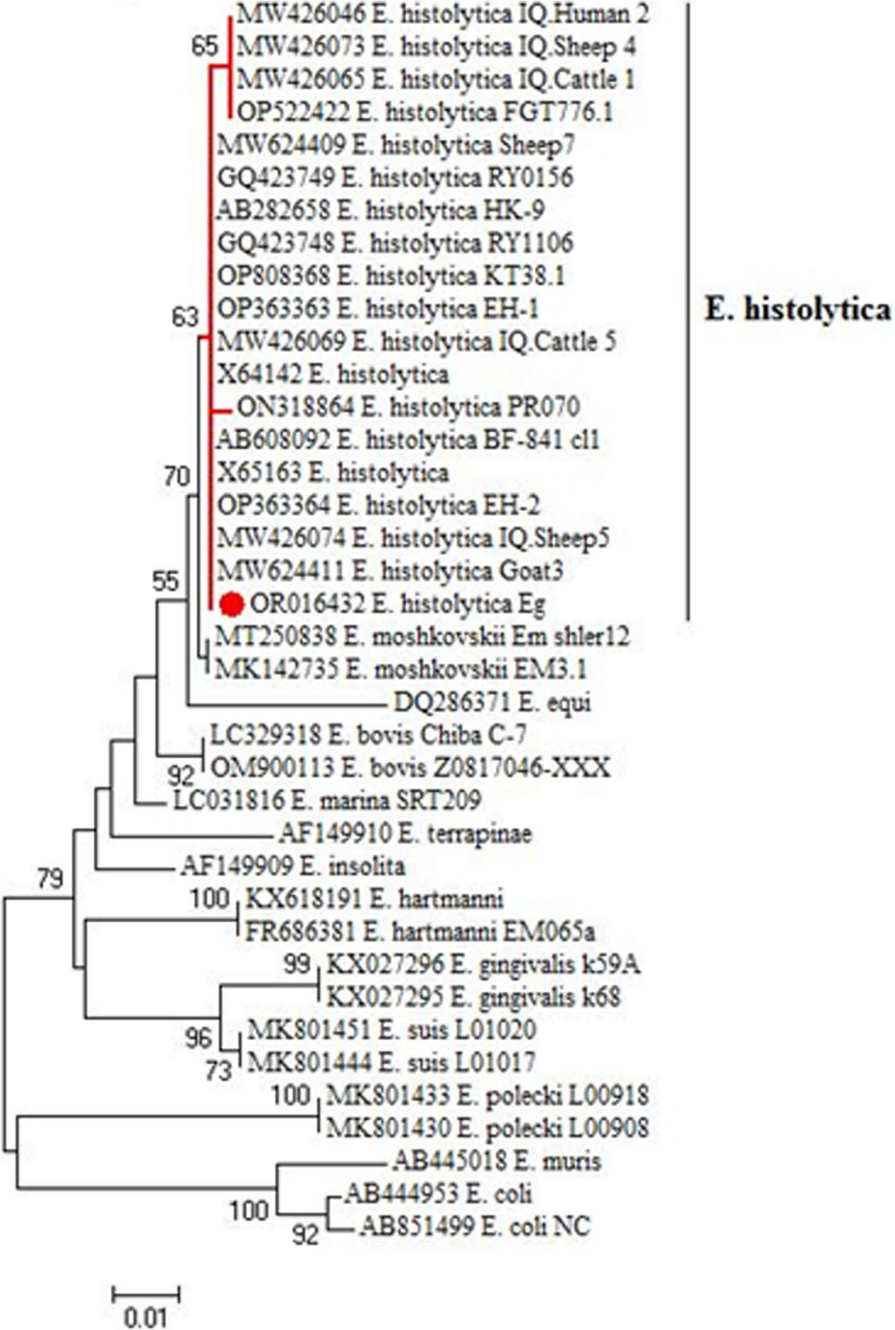
Phylogenetic analysis using the maximum likelihood method based on *ssu rRNA* gene for *Entamoeba* sp. showing a well-defined clustering of the study isolate (red circle) within the *E. histolytica* clade

*Entamoeba* DNA was detected in the fecal samples of heavily infected sheep. The nucleotide BLAST search revealed one genotype which is *E. histolytica* isolated from sheep feces from Giza and its sequence was submitted to the GenBank with the number: (GenBank: OR016432.1). Phylogenetic analysis of the *ssu rRNA* gene confirmed that the sequenced isolate clustered within the *E. histolytica* clade with high confidence and was clearly separated from other *Entamoeba* spp., supporting the molecular identity of the isolate used for antigen preparation (Fig. 2).

### Protein content

CGAg protein content was 5 mg/mL, while it was 6 mg/mL for CEAg. The total protein content of anti-*Giardia* IgG pAbs and anti-*Entamoeba* IgG pAbs produced in rabbit serum was 8.5 mg/mL and 7 mg/mL, respectively, and dropped to 4.5 mg/mL and 3 mg/mL after purification steps, respectively.

### Immunogenic responses of anti-*Giardia *IgG and anti-*Entamoeba* IgG antibodies in rabbit sera

**Fig. 3:**
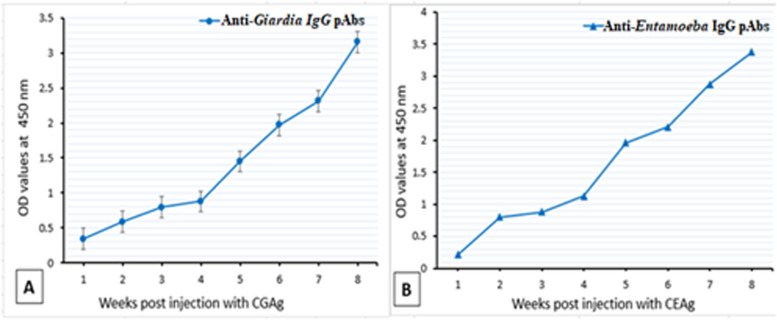
Time based rabbit antibody response to crude *Giardia* antigen (CGAg) and crude *Entamoeba* antigen (CEAg), and the potency of the two IgG polyclonal antibodies in reacting with CGAg (**A**) and CEAg (**B**) by indirect ELISA

Antibody levels increased after 1 week of the 1 st booster dose and continued to increase after the last booster dose until reaching the highest titer of antibody against CGAg and CEAg at OD values 3.158 ± 0.102 and 3.375 ± 0.151 at 1/100 dilution using indirect ELISA, respectively. The two polyclonal antibodies produced showed strong reactivity against CGAg and CEAg, as shown in Fig. 3 A & B.

### The immunodiagnostic performance of the purified anti‑*Giardia* IgG and anti-*Entamoeba *IgG pAbs in the detection of *Giardia *and *Entamoeba* antigens in animal fecal samples by Sandwich ELISA

**Fig. 4:**
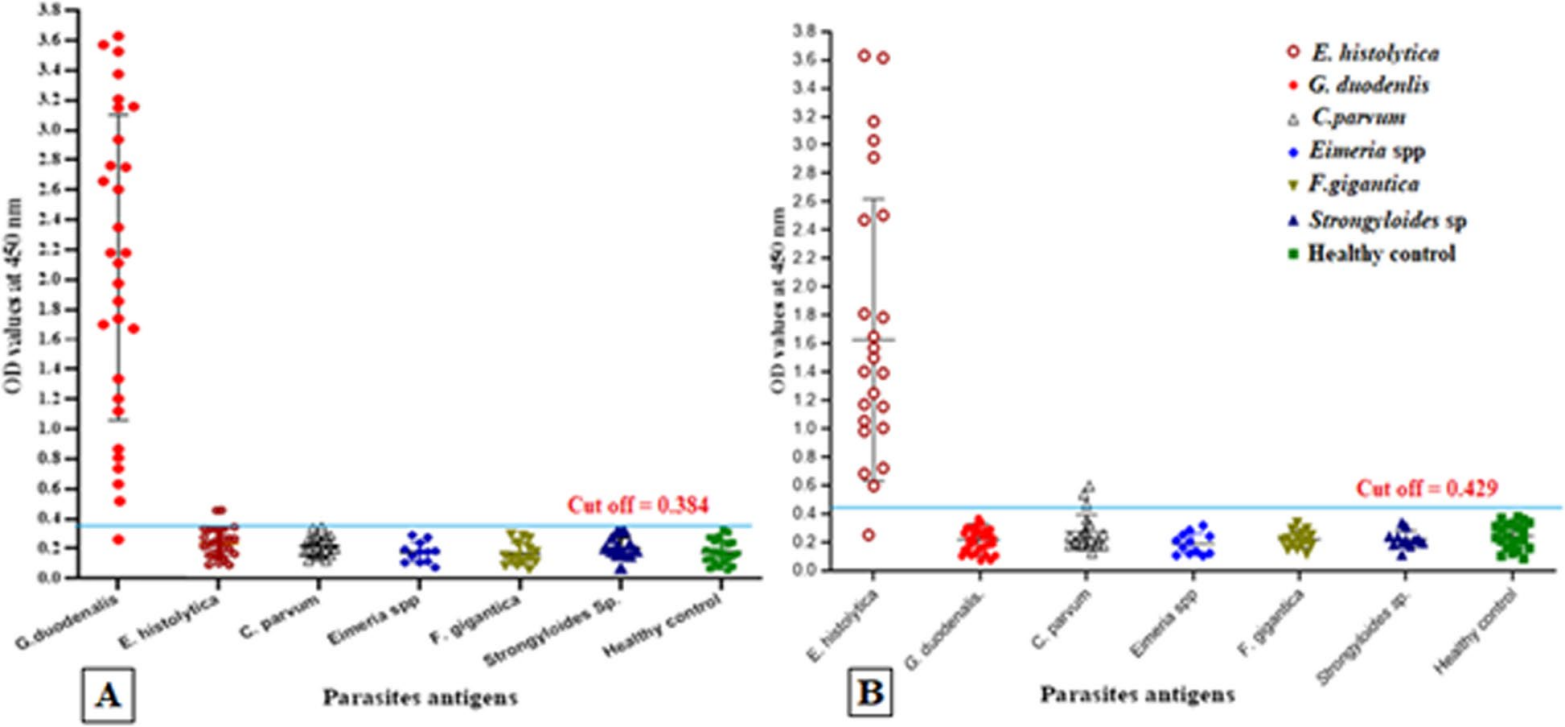
Comparative immunodiagnostic performance of purified anti‑*Giardia* IgG (**A**) and anti-*Entamoeba* IgG (**B**) polyclonal antibodies in the detection of *Giardia* and *Entamoeba* antigens in fecal samples of infected animals by sandwich ELISA

Detection of *Giardia* cyst antigens from fecal samples showed a mean OD value of 2.081 ± 1.004 which was higher than the cut-off value (0.384); and higher than those of the healthy animals (0.2040 ± 0.078) and other parasites’ samples (0.266 ± 0.093, 0.249 ± 0.065, 0.206 ± 0.066, 0.197 ± 0.073 and 0.233 ± 0.069 for *E. histolytica*, *C. parvum*, *Eimeria* sp., *F. gigantica*, and *Strongyloides* sp., respectively) as shown in Fig. 4 A. One sample out of *Giardia* infected samples showed false-negative results to record SN of 96.7%, while two out of other parasites’ samples showed false-positive results recording 98.09% SP, 93.5% PPV and 94.44% NPV. Whereas the CR was recorded 2.4%, and 97.8% DE, as shown in Table 3.

**Table 3:** Diagnostic parameters for the two purified IgG polyclonal antibodies assayed by sandwich ELISA

Diagnostic parameter	Se	Sp	AUC	CR	DE	NPV	PPV
Antibody							
Anti‑*Giardia* IgG pAbs	96.7%	98.09%	0.974^*^	2.4%	97.8%	94.44%	93.5%
Anti-*Entamoeba* IgG pAbs	95.8%	97.1%	0.965^*^	3.6%	96.89%	99.03%	88.5%

*IgG* Immunoglobulin G, *pAbs* polyclonal antibodies, *Se* Sensitivity, *Sp* Specificity, *AUC* Area under curve, *CR* Cross-reactivity, *DE* Diagnostic efficiency, *NPV* Negative predictive value, *PPV* Positive predictive value

^*^p < *0.05*

In the detection of *Entamoeba* antigens, cut off value for positivity was 0.429 (Fig. 4: B), the mean OD values of *Entamoeba* infected samples were 1.650 ± 1.005 which was higher than both samples from healthy animals (0.243 ± 0.088) and samples infected by other parasites (0.220 ± 0.092). Only one sample of positive *Entamoeba* infected samples showed false-negative result, and the SN of the assay was 95.8%. All negative control samples were below the cut-off value, while 3 fecal samples of *C. parvum* infected samples showed false-positive results recording 97.1% SP. The PPV and NPV were 88.5% and 99.03%, respectively. While the CR and DE were 3.6% and 96.89%, respectively (Table 3). The in-house sandwich ELISA demonstrated excellent diagnostic performance. ROC analysis showed outstanding discrimination between positive and negative samples, with AUC values of 0.974 for the anti-*Giardia* assay and 0.965 for the anti-*Entamoeba* assay. Diagnostic sensitivity exceeded 95% for both assays, while specificity remained above 97%, indicating high analytical accuracy with minimal cross-reactivity toward other common gastrointestinal parasites as shown in Table 3.

### Detection of *Giardia and Entamoeba* antigens in animal fecal samples by sandwich ELISA

In this study, using sandwich ELISA, 1208 (45.2%) out of 2674 fecal samples tested were found positive for *Giardia* sp. with infection rates of 68.1% (354/520) in sheep, 63.3% (322/509) in goats, 26.2% (229/875) in buffaloes, and 39.4% (303/770) in cattle. In addition, 805 out of 2674 (30.1%) samples were found positive for *Entamoeba* sp. with infection rates of 47.9% (249/520) in sheep, 37.3% (190/509) in goats, 24.8% (217/875) in buffaloes, and 19.4% (149/770) in cattle. A significant difference was observed between conventional microscopic examination and sandwich ELISA in the detection of *Giardia* spp. (*χ*^*2*^ = 281.2, *p* < *0.001*) and *Entamoeba* spp. (*χ*^*2*^ = 125.6, *p* < *0.001*). In both cases, sandwich ELISA demonstrated significantly higher detection rates compared to microscopy as summarized in Table 4. The highly significant Chi-square values (χ^2^ = 281.2 for *Giardia* and χ^2^ = 125.6 for *Entamoeba*, *p* < *0.001*) indicate that the differences between the two diagnostic methods were unlikely to have occurred by chance. These results demonstrate that sandwich ELISA detected substantially more positive samples than conventional microscopy across all examined animal species.

**Table 4:** Comparison between conventional microscopic examination and Sandwich ELISA for the diagnosis of *Giardia* and *Entamoeba* infection in farm animals

Parasite species	*Giardia*		*Entamoeba*	
Technique	Conventional Microscopy	Sandwich ELISA	Conventional Microscopy	Sandwich ELISA
Animal species				
Buffaloes (*N* = 875)	99 (11.3%)	229 (26.2%)	118 (13.5%)	217 (24.8%)
Cattle (*N* = 770)	142 (18.4%)	303 (39.4%)	73 (9.5%)	149 (19.4%)
Sheep (*N* = 520)	232 (44.6%)	354 (68.1%)	150 (28.8%)	249 (47.9%)
Goats (*N* = 509)	153 (30.1%)	322 (63.3%)	116 (22.8%)	190 (37.3%)
Total	626 (23.4%)	1208 (45.2%)	457 (17.1%)	805 (30.1%)
*ꭓ2*	281.2^*^		125.6^*^	

^*^p < 0.001

## Discussion

*Giardia* and *Entamoeba* species infect both animals and humans worldwide [6, 7]. In the current study, using microscopy, *Giardia* and *Entamoeba* cysts were detected in 23.4% and 17.1% of farm animals samples examined, respectively. Our results were comparable to previous studies in which the average prevalence of *G. duodenalis* infection was 5% (34/678) by microscopy after Lugol’s iodine staining, with 5.6% (30/539) infection rate in sheep versus 2.9% (4/139) in goats [84]. *Entamoeba* infection rate was 6.4% [11] and 60% [56] in goats, 36% in sheep [42], and 43% [53] and 72% [42] in cattle, while Ai et al. [7] recorded severe *Entamoeba* sp. infection (100%) in goats, sheep and cattle. The higher infection rate observed in the livestock might be due to environmental conditions, population density, poor sanitation, lack of implementation of prevention measures [7, 45] and the simple transmission pattern and zoonotic nature of these protozoans making them ready to spread to humans and non-human primates [31]. In the current study, we found marked variations in the reported infection rates of *Giardia* and *Entamoeba* spp. in cattle, buffaloes, sheep and goats, where young animals were more vulnerable to infections. These variations might be due to the age of the animals, their management practices, housing, feeding, and the detection methods used [64]. As the age of animals increases, cyst excretion decreases and becomes intermittent [35, 82] probably due to limited functional integrity of the immune system or slow development of specific immunity by animal against these parasites [74].

Diagnosis of protozoans is usually conducted by fecal microscopy, particularly in low-resource settings [24], which remains the conventional diagnostic method but its sensitivity is relatively low even after multiple examinations [43]. Most studies focus on single-parasite infections, despite growing evidence that GI parasitic infections might predispose co-infections which could exhibit synergistic effects on the disease severity and treatment outcomes [21]. In our study, there was an association between *Giardia* and *Entamoeba* species with other parasitic infections in farm animals as reported in some earlier studies [26, 39, 63, 77] and this might be due to contamination of animal surrounding environment and water resources.

*G. duodenalis* (synonyms: “*G. lambilia*” and “*G. intestinalis*”) comprises eight assemblages (A–H), in which assemblages A and B exhibit low host specificity and infect humans and a diverse range of animals [64]. The phylogenetic analyses of the obtained *Giardia* and *Entamoeba* isolates demonstrated a strong concordance with the previously deposited sequences in GenBank, particularly those identified in humans and animal hosts from Egypt. The clustering of our *G. duodenalis* isolates within Assemblage A were closely related to Egyptian reference sequences (GenBank: HM171702.1, MG746615.1, KP899830.1, MG736240.1, PP035399.1, and MG736240.1), and aligned with *G. duodenalis* Assemblage A identified in cattle from Greece (GenBank: MK452850.1) and humans from Kenya (GenBank: LC508625.1), Ethiopia (GenBank: KP899830.1, MG736240.1 and PP035399.1), Brazil (GenBank: MG807894.1) and Iraq (GenBank: PX646761.1) with high sequence similarity and limited polymorphism. The proximity of our study isolates to human-derived Egyptian sequences, rather than to animal-specific assemblages such as Assemblage E, suggests shared environmental contamination or possible cross-species transmission [6, 37]. In addition, *G. duodenalis* Assemblage B were detected in this study in cattle and goat and were identical to those isolated from humans from Egypt (GenBank: MG736247.1 and KR260646.1). The phylogenetic analysis demonstrated that all *G. duodenalis* isolates obtained in this study clustered within the zoonotic Assemblage A, showing close genetic relatedness to isolates previously reported from both humans and livestock in Egypt and several other countries. This finding indicates that genetically similar strains circulate among different host species and supports the presence of genotypes with broad host ranges rather than strict host specificity. Likewise, the *Entamoeba* isolate clustered confidently within the *E. histolytica* clade, confirming the molecular identity of the isolates used for antigen preparation. Although phylogenetic similarity alone cannot determine the direction of transmission between humans and animals, the close relationship between animal-derived isolates and published human isolates suggests shared transmission cycles or common environmental contamination. These findings highlight the epidemiological importance of molecular surveillance for identifying circulating genotypes and monitoring parasites with zoonotic potential within a One Health framework. Our results agree with many studies that recorded assemblages A and B in farm animals: cattle, buffaloes and sheep [20, 28, 59, 63]. Similarly, the tight clustering of the study isolates with GenBank sequences derived from both human and animal sources indicate a high degree of genetic conservation across hosts [65]. In our study, sequencing was performed on a limited number of representative isolates selected for antigen preparation rather than on all microscopy- or ELISA-positive samples. Therefore, the phylogenetic findings describe the circulating genotypes identified in the sequenced isolates but do not represent the full genetic diversity of *Giardia* or *Entamoeba* infections in the study population. Future studies using larger numbers of sequenced isolates would further clarify genotype distribution and transmission dynamics.

In our study, the overall infection rate was nearly doubled for (23.4% by microscopy vs. 45.2% by sandwich ELISA) and markedly increased for amoebiasis (17.1% by microscopy vs. 30.1% by sandwich ELISA), indicating that microscopy alone may underestimate the true prevalence of these infections. This pattern was consistent across buffaloes, cattle, sheep, and goats, with the highest infection rates were observed in small ruminants, particularly sheep. These results agreed with recent studies evincing the superior sensitivity of coproantigen-based tests [3, 29, 77]. In contrast, microscopy is inherently limited by operator dependency and low sensitivity in subclinical or low-intensity infections [5, 66]. Sensitivity and specificity depend on many factors, such as the laboratory techniques used and the antigen quality, stability, and source [4]. Developing a reliable coprological diagnosis in farm animals in the form of antigen detection technique using the standard sandwich ELISA might be a promising alternative to detect fecal *Giardia* and *Entamoeba* antigens with high sensitivity and specificity, simple and cost-effective for use in developing countries where these diseases are prevalent [3, 16] and to distinguish the pathogenic *E. histolytica* from other *Entamoeba* species [81]. Apparently, there is no commercial microtiter plate-format ELISAs available for diagnosis of giardiasis and amoebiasis in animal samples [10, 22, 60, 62, 72], however, some veterinarians have reported success with those designed for human samples using dog samples [75]. Polyclonal antibodies are often preferred during the initial development of immunodiagnostic assays because their ability to recognize multiple epitopes increases the likelihood of detecting soluble antigens present at low concentrations or after partial antigen degradation in fecal samples. In our study, we used the purified cyst antigens of *G. duodenalis* and *E. histolytica* to produce polyclonal antibodies to produce an in-house sandwich ELISA for the detection of *Giardia* and *Entamoeba* antigens with significantly high sensitivity and specificity (*p* < *0.001*). The better performance of the sandwich ELISA produced in the current study might be attributed to its ability to detect soluble protozoan antigens independently of cyst integrity and/or uneven parasite distribution in feces compared to microscopy which relies heavily on the expertise of the microscopist and the fecal sample quality [33]. In addition, the higher detection rates obtained by sandwich ELISA in this study might reflect improved specificity for pathogenic antigens compared to microscopy where pathogenic *E. histolytica* are morphologically indistinguishable from non-pathogenic species [17, 30]. In our study, the significantly higher positivity rates obtained by the sandwich ELISA compared with microscopy may indicate improved detection of coproantigens in samples with low parasite burden or intermittent cyst shedding, which are commonly missed by microscopic examination. However, because microscopy was used as the comparative reference method, it cannot be excluded that some additional ELISA-positive results represent false-positive reactions. Nevertheless, the assay demonstrated high analytical specificity, minimal cross-reactivity with other common gastrointestinal parasites, and excellent ROC performance (AUC > 0.96), supporting its reliability as a screening tool. Further studies comparing the assay against comprehensive molecular testing of all samples would provide a more definitive assessment of its diagnostic accuracy. This highlights the continued need for molecular confirmation, particularly in studies aiming to assess zoonotic risk or genotype distribution. Antigen screening ELISA succeeded in detecting *Giardia* coproantigens [67] and is recommended for its ability to monitor acute infections with high sensitivity and specificity, especially in large animal farms using monoclonal antibodies (mAbs) and pAbs [46], than microscopical methods [32]. Many studies using *E. histolytica* antigen detection tests have shown sensitivity for the *E. histolytica* antigen detection to range from 80 to 94% [54]. When microscopy was the reference technique, *Giardia* and *Entamoeba* antigen testing offered better and reliable sensitivity and specificity [9, 41, 48]. In addition, it was found that combined use of rapid tests and microscopic examination increased the sensitivity of detection [81]. The high positive and negative predictive values recorded in our study further indicate that positive ELISA results are highly likely to represent true infections, while negative results reliably exclude infection under the study conditions. Together with the high diagnostic efficiency (> 96%), these findings demonstrate the practical applicability of the developed assay for routine veterinary diagnosis. In addition, in our study, the use of polyclonal antibodies might introduce batch variability and nonspecific binding. Future studies should investigate monoclonal antibody-based assays for improved standardization and specificity. Although the assay showed excellent diagnostic performance in the present study, validation was performed using samples collected in Egypt. Additional multicenter validation involving larger numbers of PCR-confirmed positive and negative samples from different geographical regions would further establish its reproducibility and general applicability.

## Conclusion

In our study, from a One-Health perspective, the high prevalence of *Giardia* and *Entamoeba* infections in livestock underscores their potential role as reservoirs for zoonotic transmission. This study supports the adoption of a developed sandwich ELISA which is a sensitive diagnostic approach for *Giardia* and *Entamoeba* infections in farm animals. We recommend integration of this antigen detection assay into routine diagnostic protocols to improve accuracy along with complementary molecular tests to confirm species identity and assess zoonotic risk.

## Supplementary Information

Supplementary Material 1: Supplement. Table 1: Survey of gastrointestinal parasites infection rates according to host species and age group of animals.

Supplementary Material 2.

Supplementary Material 3.

## Data Availability

All data generated and analyzed in this study are included in the published manuscript. The nucleotide sequences of G. duodenalis for the beta-giardin gene and E. histolytica for the ssu rRNA gene obtained in this study have been submitted to the GenBank with accession numbers: OQ388331.1, OQ388332.1, OR029977.1, OR029976.1, OR029974.1, OR029975.1, PP316110.1 and OR016432.1 (https://www.ncbi.nlm.nih.gov/genbank).

## Abbreviations

AUC: Area Under Curve
*Bg*: *β-giardin*
CEAg: Crude *Entamoeba* antigen
CGAg: Crude *Giardia* antigen
CI: Confidence Interval
CR: Cross reactivity
DE: Diagnostic efficiency
*E.*: *Entamoeba*
ELISA: Enzyme Linked Immunosorbent Assay
*G.*: *Giardia*
HRP: Horseradish Peroxidase
IgG: Immunoglobulin G
Kg: Kilograms
mL: Milliliters
MZN: Modified Ziehl–Neelsen
NPV: Negative Predictive Value
OD: Optical Density
OPD: *O*-Phenylenediamine-dihydrochloride
pAbs: Polyclonal antibodies
PBS: Phosphate Buffered Saline
PCR: Polymerase Chain Reaction
PPV: Positive Predictive Value
ROC: Receiver Operating Characteristic
SD: Standard deviation
Se: Sensitivity
Sec: Seconds
Sp: Specificity
spp.: Species
*ssu rRNA*: Small subunit ribosomal RNA
μg: Micrograms
