# Supplementary material for: Giardia duodenalis and Entamoeba spp.: Coprological and antibody–based approaches across farm animals in Egypt
Source: BMC Vet Res. 2026 Aug 14;22:475. doi: 10.1186/s12917-026-05801-4 (PMC13476928; doi:10.1186/s12917-026-05801-4)
Supplement: Supplementary file 1 [file 12917_2026_5801_MOESM1_ESM.docx]

**Supplement. Table 1**: Survey of gastrointestinal parasites infection rates according to host species and age group of animals.

| **GI parasite**  **species**  **Animal**  **species/age** | | **Protozoan Parasitic Infections** | | | | **Helminth Parasitic Infections** | | | | |
| --- | --- | --- | --- | --- | --- | --- | --- | --- | --- | --- |
|  |  | ***Giardia*** | ***Entamoeba*** | ***Cryptosporidium*** | ***Eimeria*** | ***Toxocara vitulorum*** | ***Strongyloides*** | ***Moniezia*** | ***Fasciola*** | ***Trichuris*** |
| **Buffaloes** | **> 4 months**  **(N= 88)** | 34 (38.6%) | 27 (30.7%) | 32 (36.4%) | 29 (33%) | 18 (20.5%) | 0 | 0 | 0 | 0 |
|  | **4-6 months**  **(N = 122)** | 29 (23.8%) | 25 (20.5%) | 41 (33.6%) | 37 (30.3%) | 39 (32%) | 3 (2.5%) | 0 | 0 | 0 |
|  | **6-12 months**  **(N= 180)** | 16 (8.9%) | 22 (12.2%) | 48 (26.7%) | 35 (19.4%) | 58 (32.2%) | 16 (8.9%) | 0 | 3 (1.7%) | 0 |
|  | **≥ 1 year**  **(N = 485)** | 20 (4.1%) | 44 (9.1%) | 78 (16.1%) | 84 (17.3%) | 87 (17.9%) | 118 (24.3%) | 0 | 187 (38.6%) | 82 (16.9%) |
|  | **Sub-total**  **N=875** | **99 (11.3%)** | **118 (13.5%)** | **199 (22.7%)** | **185 (21.1%)** | **202 (23.1%)** | **137 (15.7%)** | **0** | **190 (21.7%)** | **82 (9.4%)** |
| **Cattle** | **> 4 months**  **(N= 88)** | 35 (39.8%) | 26 (29.5%) | 29 (33%) | 32 (36.4%) | 35 (39.8%) | 0 | 0 | 0 | 0 |
|  | **4-6 months**  **(N = 122)** | 56 (45.9%) | 33 (27%) | 57 (46.7%) | 41 (33.6%) | 47 (38.5%) | 12 (9.8%) | 0 | 0 | 0 |
|  | **6-12 months**  **(N = 190)** | 28 (14.7%) | 9 (4.7%) | 48 (25.3%) | 35 (18.4%) | 63 (33.2%) | 10 (5.3%) | 0 | 2 (1.1%) | 0 |
|  | **≥ 1 year**  **(N = 350)** | 23 (6.6%) | 5 (1.4%) | 79 (22.6%) | 24 (6.9%) | 45 (12.9%) | 85 (24.3%) | 0 | 92 (26.3%) | 12 (3.4%) |
|  | **Sub-total**  **N=770** | **142 (18.4%)** | **73 (9.5%)** | **213 (27.7%)** | **132 (17.1%)** | **190 (24.7%)** | **107 (13.9%)** | **0** | **94 (12.2%)** | **12 (1.6%)** |
| **Sheep** | **Lambs**  **(N = 160)** | 84 (52.5%) | 61 (38.1%) | 27 (16.9%) | 50 (31.3%) | 0 | 28 (17.5%) | 13 (8.1%) | 0 | 3 (1.8%) |
|  | **Sheep**  **(N = 360)** | 148 (41.1%) | 89 (24.7%) | 40 (11.1%) | 71 (19.7%) | 0 | 156 (43.3%) | 117 (32.5%) | 73  (20.3%) | 25  (6.9%) |
|  | **Sub-total**  **N=520** | **232 (44.6%)** | **150 (28.8%)** | **67 (12.9%)** | **121 (23.3%)** | **0** | **184 (35.4%)** | **130 (25%)** | **73 (14%)** | **28 (5.4%)** |
| **Goats** | **Goat kids**  **(N = 158)** | 70 (44.3%) | 46 (29.1%) | 17 (10.8%) | 52 (32.9%) | 0 | 14 (8.9%) | 37 (23.4%) | 6 (3.8%) | 0 |
|  | **Goat**  **(N = 351)** | 83 (23.6%) | 70 (19.9%) | 30 (8.5%) | 78 (22.2%) | 0 | 95 (27.1%) | 67 (19.1%) | 38 (10.8%) | 10 (2.8%) |
|  | **Sub-total**  **N=509** | **153 (30.1%)** | **116 (22.8%)** | **47 (9.2%)** | **130 (25.5%)** | **0** | **109 (21.4%)** | **104 (20.4%)** | **44 (8.6%)** | **10 (2%)** |
